# Adolescent sports participation and social-class health inequalities: evidence from CHNS

**DOI:** 10.3389/fpubh.2026.1791550

**Published:** 2026-05-15

**Authors:** Jie Yu, Yao Lu, Lunan Zhao

**Affiliations:** 1 Department of College English Teaching, Qufu Normal University, Qufu, Shandong, China; 2 College of Physical Education and Sport Science, Qufu Normal University, Qufu, Shandong, China

**Keywords:** adolescents, class inequality, health, life course, sports participation

## Abstract

**Purpose:**

Health inequalities rooted in social stratification remain a persistent structural public-health challenge. While physical activity is recognized as a health-enhancing behavior, limited longitudinal evidence exists on how its effects vary across social classes. This study examines the life-course interactions among social class, physical-activity participation, and aging, and evaluates whether youth physical activity mitigates class-based health disparities.

**Methods:**

Data were drawn from five waves (1997, 2000, 2004, 2006, and 2015) of the China Health and Nutrition Survey (CHNS), comprising 39,672 individuals and 180,696 person-year observations. Growth-curve models were used to estimate trajectories of self-rated health and assess the interactive effects of social class, physical activity, and age, controlling for demographic characteristics.

**Results:**

Health inequalities exhibited structural rigidity: health gradients between social strata widened from 1997 to 2015, and early-life disparities persisted throughout aging, forming “parallel trajectories.” Physical activity effects differed significantly by class. Lower-stratum individuals benefited little due to structural constraints; middle-stratum participants showed a “health-behavior paradox,” where participation correlated with steeper health decline; upper-stratum groups demonstrated negligible marginal gains due to resource saturation.

**Conclusion:**

Universal sports-promotion strategies are insufficient to reduce entrenched health inequalities. Structural reforms addressing socioeconomic constraints, combined with class-sensitive behavioral interventions, are essential to disrupt the reproduction of health disparities and promote equitable healthy aging across the life course.

## 1 Introduction

Health disparities arising from socioeconomic inequality have become one of the most persistent structural challenges in global public health (1, 2). Substantial evidence demonstrates that across both developed and developing societies, individuals with higher socioeconomic status (SES) consistently enjoy superior health outcomes, whereas those from resource-deprived backgrounds are more vulnerable to poor physical conditions, heightened psychological stress, and insufficient physical activity (3). Addressing how this health stratification can be mitigated remains a critical question. Against this backdrop, adolescent sports participation has attracted increasing scholarly attention as a behavioral factor shaping long-term health trajectories (4). Adolescence represents a pivotal period for the accumulation of health capital, the formation of behavioral habits, and the development of socio-emotional competencies. Regular physical activity not only promotes physiological development but also fosters long-term well-being through mechanisms such as stress regulation, social support, and behavioral self-efficacy (5). However, most existing studies emphasize the overall benefits of physical activity while neglecting its distributional effects—namely, whether participation narrows health disparities across social strata or instead amplifies them as a new mechanism of inequality reproduction (6). Moreover, prior research has largely relied on cross-sectional designs and Western samples. Longitudinal evidence remains limited regarding how adolescent physical activity affects health inequalities in China’s context of rapid social transformation and intense educational competition. To fill these gaps, this study adopts a life course perspective and employs longitudinal data from the China Health and Nutrition Survey (CHNS, 1997–2015) to examine whether and how adolescent sports participation influences the formation and evolution of health stratification. The life course framework highlights how early-life experiences accumulate and shape long-term health trajectories, emphasizing both the compensatory and reinforcing roles of behavioral exposures. Within this framework, this study investigates whether adolescent physical activity functions as a compensatory mechanism that mitigates socioeconomic disadvantage, or a reinforcing mechanism that strengthens health inequalities (7, 8). The findings aim to enrich empirical understanding of the intergenerational transmission of health inequality and identify potential policy intervention windows.

### 1.1 Cumulative disadvantage hypothesis

Health disparities across social classes are not static reflections of current living conditions but dynamic outcomes of accumulated disadvantages over time (9). The Cumulative Disadvantage Hypothesis, posits that early socioeconomic disadvantages compound throughout the life course, leading to increasingly divergent health trajectories. Individuals from low-SES backgrounds face heightened health risks not only due to material deprivation but also because of limited access to key institutional resources such as education, healthcare, and social support (10). Case et al. (11) found that income-related health disparities emerge during adolescence and widen with age, suggesting that socioeconomic disadvantage is self-reinforcing. Hertzman and Boyce (12) further emphasize that early-life conditions “get under the skin,” embedding inequality at the biological level through stress and behavioral pathways. These insights underscore that health inequality reflects a cumulative process shaped by structural constraints and life-course exposure. Hypothesis 1 (H1): Health inequalities attributable to social class will progressively increase with age.

### 1.2 The age-neutralizing effect hypothesis

Life course research suggests that the development of health inequalities is not always a linear process of cumulative disadvantage but may exhibit an age-dependent neutralizing effect. This hypothesis posits that health disparities rooted in early socioeconomic differences may gradually narrow in later life as various social mechanisms interact to mitigate inequality (13). Rather than continuously widening, health trajectories between groups may converge or stabilize over time. Kim et al. (14) attribute this phenomenon partly to selective survival, whereby individuals with poorer early-life health are less likely to remain in later samples, producing a more homogeneous health profile among survivors. Zhou et al. (15) argue that universal access to healthcare, public health improvements, and widespread education collectively generate a leveling effect during adulthood, reducing baseline disparities across socioeconomic strata. Framed within this life course perspective, Hypothesis 2 (H2) proposes that health inequalities attributable to social stratification will gradually diminish with age.

### 1.3 Critical period effects and institutional compensation

Life course theory underscores the phased nature of individual responses to environmental influences. The critical period and sensitive period hypotheses posit that behavioral exposures during specific developmental stages exert disproportionately strong—and sometimes irreversible—effects on long-term health trajectories (16). Adolescence represents such a plastic phase, characterized by physiological maturation, psychological reorganization, and the consolidation of lifestyle habits. Within this window, engagement in physical activity can shape early adult health and produce cumulative advantages over time (17). This temporal framework provides a basis for examining whether adolescent sports participation can modify the developmental pathways of health inequality. Complementarily, the institutional compensation hypothesis argues that public institutions such as schools and community programs can offset socioeconomic disadvantages by offering universal and accessible resources (18). Applied to sports, institutionalized participation opportunities may yield higher marginal returns for resource-deprived adolescents, creating a compensatory effect. Conversely, when access to sports depends heavily on family capital, higher-status groups may capture greater benefits, producing a reinforcing effect. Grounded in these theoretical perspectives, this study advances Hypothesis 3 (H3): Adolescent behaviors influence health stratification across the life course.

### 1.4 Health benefits of physical activity participation from a life course perspective

Within the life course framework, health is conceptualized as a multidimensional dynamic system encompassing physical, psychological, and social dimensions, shaped by the long-term interplay between behavioral exposures and social structures (19). Physical activity, as a core health behavior, exerts sustained protective effects across developmental stages. Physiologically, it enhances cardiorespiratory fitness, muscular strength, and metabolic regulation, reducing risks of chronic disease and physical decline (20). Psychologically, regular exercise improves emotional regulation, resilience, and stress recovery, offering particular protection for adolescents exposed to social disadvantage (21). Socially, sports participation strengthens peer relationships, teamwork, and self-efficacy, fostering social capital that supports positive health behaviors even in adverse environments (22). Longitudinal research highlights a strong tracking effect of physical activity: individuals active during adolescence are more likely to sustain participation into adulthood, enabling the accumulation of long-term health advantages (23). This aligns with health investment theory, which views health as a durable capital stock yielding lifelong returns, with adolescent investment producing the greatest marginal benefits. Accordingly, Hypothesis 4 (H4) posits that although health levels decline with age, participation in physical activity mitigates this age-related deterioration.

### 1.5 The hypothesis of adolescent sports participation mitigating health inequalities across social classes

**Figure 1:**
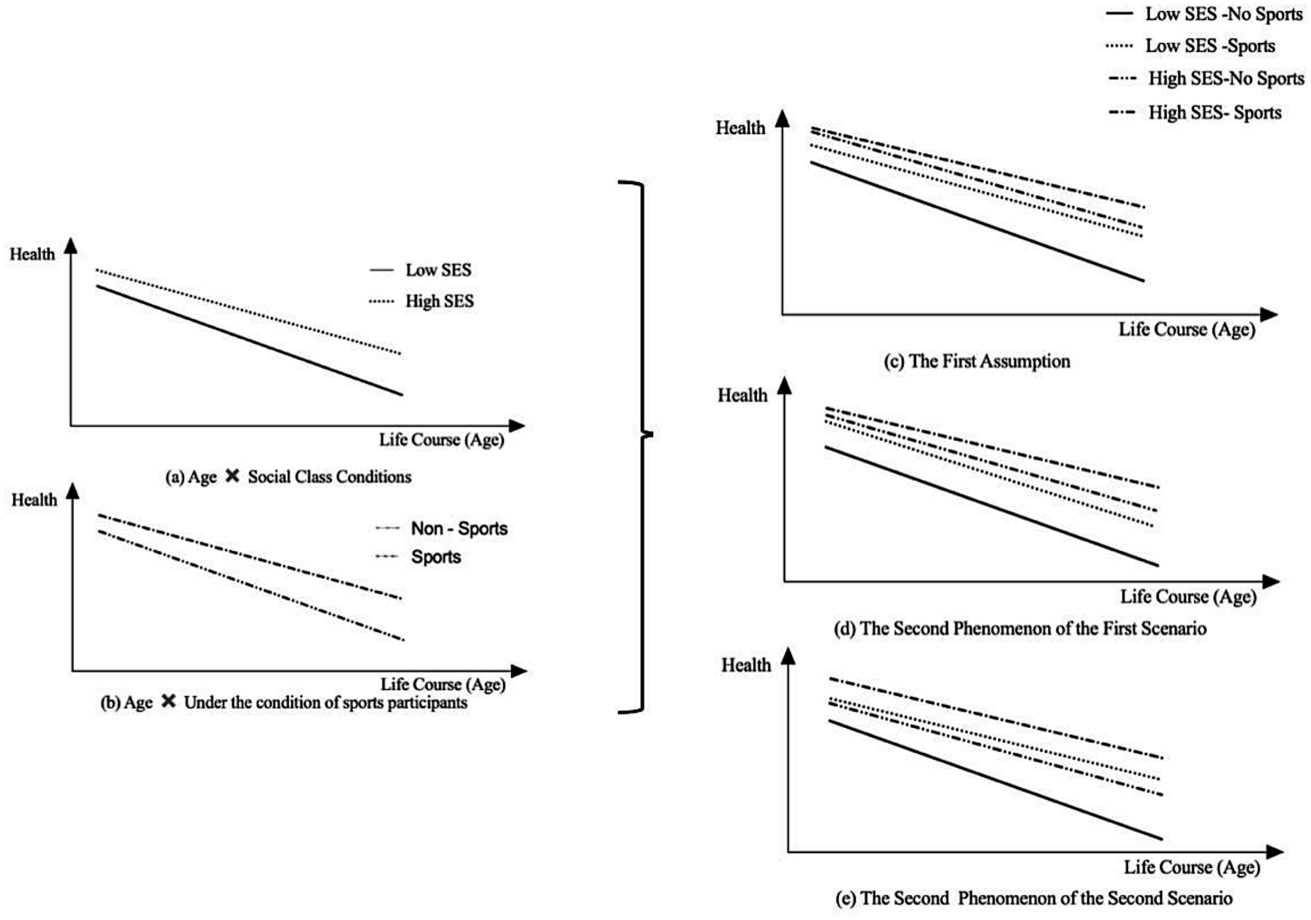
The concept of physical education breaking down health inequality among social classes. **(a)** Health trajectories by SES, showing persistent inequality between low- and high-SES groups. **(b)** Health trajectories by sports participation, showing different age-related declines. **(c)** Sports participation protects health but does not eliminate class-based inequalities. **(d)** Sports participation narrows class-based health inequalities and promotes convergence. **(e)** Sports participation reverses class-based health inequalities.

Based on theoretical analyses of health stratification inequality (Figure 1a) and the health returns from sports participation (Figure 1b), this study proposes the following hypotheses.

(1) Adolescent sports participation fails to bridge health inequalities across socioeconomic strata. In this scenario, adolescents from lower socioeconomic backgrounds who consistently engage in sports between ages 12 and 18 experience a slower decline in health with age. However, this behavioral advantage remains insufficient to offset the health disparities rooted in socioeconomic status. Even with stable exercise habits established during adolescence, individuals from low-SES groups maintain significantly lower health levels in later life than their high-SES counterparts who did not consistently participate in sports (*p* < 0.05, Figure 1c). Thus, adolescent sports participation exerts a protective but limited effect, primarily slowing the widening of health inequalities rather than eliminating structural disparities.
  **Hypothesis 5 (H5):** Sports participation during adolescence does not eliminate class-based health inequalities as individuals age.
(2) Adolescent sports participation can mitigate or reverse health inequalities across socioeconomic strata. In this scenario, adolescents from lower socioeconomic backgrounds who consistently engage in physical activities exhibit a slower health decline across the life course. Compared with their high-SES peers who do not participate in sports, two outcomes are possible. First, although the high-SES group starts with better baseline health, the lower-SES group’s sustained activity leads to a smaller health decline, gradually narrowing the gap until it becomes statistically insignificant (*p* > 0.05, Figure 1d). Second, the sustained benefits of adolescent physical activity may eventually result in lower-SES individuals surpassing the health levels of inactive high-SES peers at a later stage, representing a “reversal effect” (*p* < 0.05, Figure 1e).

**Hypothesis 6 (H6):** With increasing age, adolescent sports participation reduces class-based health disparities, leading to convergence in health levels between socioeconomic groups. Hypothesis 7 (H7): With increasing age, adolescent sports participation reverses class-based health disparities, resulting in superior health among low-SES individuals compared to high-SES counterparts.

## 2 Methods

### 2.1 Variable concepts and evaluation

#### 2.1.1 Social class

Social stratification refers to the hierarchical organization of individuals or households into distinct social ranks based on their possession of social resources, resulting in relatively stable groups with similar social status (24). In China, the Research Report on Contemporary Chinese Social Stratification led by Lu Xueyi represents a seminal contribution to this field (25). Drawing on organizational, economic, and cultural resource criteria, it classifies the population into five major levels and ten strata using occupational status as the key indicator. In the Chinese context, occupational differentiation reflects not only family background, educational attainment, and economic capacity but also institutional, structural, and behavioral disparities—constituting a multidimensional manifestation of social stratification (26). This measure captures early-life structural position rather than contemporaneous economic status, making parental occupation a more stable and conceptually appropriate indicator of the adolescent social environment than income alone. Following this framework and aligning with the data structure of this study, the parental occupation with the higher social status is used to define each respondent’s social stratum. Specifically, individuals whose higher-status parent works as a lumberjack, unskilled laborer, farmer, fisherman, or hunter are categorized as Farmers and Unskilled Workers; those whose parent is a shop assistant, craftsperson, cook, waiter, or barber are classified as Skilled and Service Workers; clerical and office-support roles correspond to the Clerical Staff; teachers, editors, nurses, and similar professional occupations were classified as Junior Intellectuals; professors, doctors, architects, and engineers were classified as Senior Intellectuals; and government or corporate managers belong to the Administrators and Managers.

For analytical clarity and to ensure adequate sample representation, adjacent strata with minimal health disparities were merged into three broader categories: (1) Lower class—including Farmers and Unskilled Workers and Skilled Workers and Service Workers; (2) Middle class—including Clerical Staff and Junior Intellectuals; (3) Upper class—including Senior Intellectuals and Administrators and Managers.

#### 2.1.2 Self-perceived health

The World Health Organization states that health is not merely the absence of disease or infirmity, but rather a state of complete physical, mental, and social well-being (27). With societal progress, people’s understanding of health has shifted from the traditional notion of “health as the absence of disease” to a multidimensional holistic perspective encompassing social, psychological, and physiological dimensions (28). Currently, social science research commonly employs self-rated health indicators to measure individual health (29). This metric reflects an individual’s comprehensive perception of their past, present, and future objective physical condition and subjective psychological well-being, encompassing dimensions of social, psychological, and physical health (30). It has been validated by domestic and international studies as an effective comprehensive health assessment tool (31). Based on this, this paper selects the self-rated health indicator to evaluate individual health levels. The research question is: “Compared to other people your age, how would you rate your current health status?” Response options are: “Very poor” (1 point), “Poor” (2 points), “Fair” (3 points), “Good” (4 points), and “Very good” (5 points), with positive adjustment applied. In medical sociology, public health, and health sociology research, self-rated health is commonly used as a dependent variable in modeling. Existing studies indicate consistent analytical results whether treated as a continuous or ordinal variable. To simplify models and facilitate interpretation, most domestic scholars in population health research generally treat it as a continuous variable. This paper follows this approach, modeling self-rated health as a continuous variable.

#### 2.1.3 Physical activity participation

Physical activity participation refers to adolescents’ deliberate engagement in organized or informal sports, exercise, and recreational activities to promote physical and mental health, enrich daily life, and facilitate social interaction (32). It involves not only presence at activity venues but also cognitive involvement, emotional experience, and social interaction, and thus serves both as an important channel of socialization and a means of improving health status. In this study, physical activity participation is operationalized using CHNS data on 12–18-year-olds. Four items are used: “U99a How many times per week do you participate in physical activities before school, after school, or on weekends?”; “U99b On average, how long does each such activity session last (hours:minutes)?”; “U109 How many times per week do you participate in physical activities at school (including during class or recess)?”; and “U109a On average, how long does each such activity session last (hours:minutes)?.”To capture overall participation, in-school and out-of-school activities are combined. For each respondent, the total weekly frequency and total weekly duration of all physical activities are calculated. If the combined weekly frequency is more than two sessions or the combined weekly duration is more than 60 min—meeting either criterion—the adolescent is classified as engaging in systematic physical activity participation and coded as 1 (“participant”); otherwise, the value is 0 (“non-participant”). Because the available CHNS items do not provide sufficiently consistent information across waves to distinguish activity type, intensity, or context in a comparable way, physical activity participation was operationalized here as a broad participation indicator.

#### 2.1.4 Demographic variables

Based on prior research, health is influenced by a range of demographic characteristics, including region, gender, household registration, age, marital status, education, and income (33–35). Accordingly, this study includes region, gender, household registration, and age as control variables. Region is coded as Western = 0, Central = 1, Northeast = 2, and Eastern = 3; gender as female = 0, male = 1; and household registration as rural = 0, urban = 1. Because the data are longitudinal, age enters the models as a time-varying continuous variable, defined as respondents’ actual age in each survey wave, which is consistent with the life-course perspective adopted in this study. The social class index used here jointly reflects educational background, organizational resources, and economic resources. Income and education were not included in the final models not only because they are highly correlated with social class, but also because they overlap conceptually with class position and may partly function as downstream mediators rather than independent confounders over the life course. To examine how social class interacts with sports participation and age in shaping health outcomes, social class is treated as a continuous variable. This specification exploits its inherent ordinality, allows potential dose–response relationships with health to be captured more precisely, and reduces the number of parameters to be estimated, thereby improving model parsimony and stability. Gender, household registration, and region are also entered as 0/1 or 0–3 coded continuous variables. For binary variables, 0/1 coding is algebraically equivalent to categorical treatment, while for household registration and region it approximates underlying socioeconomic gradients in access to resources and development opportunities. Sensitivity analyses indicate that, relative to conventional dummy-variable coding, this continuous specification preserves the direction of core effects while reducing the variance of key estimates and improving model convergence, thus yielding more precise and robust parameter estimates for the interaction models.

### 2.2 Data sources

The data used in this study are drawn from the China Health and Nutrition Survey (CHNS), a long-term longitudinal project jointly conducted by the Chinese Center for Disease Control and Prevention and the Carolina Population Center at the University of North Carolina at Chapel Hill. The CHNS is widely recognized for its reliability and validity and has been extensively used in both domestic and international research. As an individually linked panel survey, it tracks the same respondents over time, with repeated observations across waves connected through unique individual identifiers, thereby providing a robust foundation for longitudinal analysis.

Given the availability of the adolescent physical-activity items used to construct the key predictor, we drew on five survey waves (1997, 2000, 2004, 2006, and 2015). The most recent publicly available CHNS data relevant to this study extend to 2015, and more recent waves are either not yet publicly available or do not provide fully comparable measures for the variables used here. After initial screening, the analytic sample comprised 39,672 individuals, yielding 180,696 person-wave observations. Despite variation in participation across waves, the resulting longitudinal sample provides a sufficiently broad basis for depicting health trajectories among Chinese adults over the life course, as illustrated by the subsample used for growth-curve modeling in Table 1.

**Table 1:** List of variable information.

Type	Variable	Coding	Percentage/%	Min	Max	SD
Control variables	Region	Western = 0	25.8	0	3	1.048
		Central = 1	38.7			
		Northeastern = 2	16.6			
		Eastern = 3	18.9			
Independent variables	Age^a^	Age in years (continuous)	100.0 (18.7)	12	37	5.099
	Gender	Female = 0	38.2	0	1	0.486
		Male = 1	61.8			
	Residency Status	Rural = 0	64.2	0	1	0.480
		Urban = 1	35.8			
	Social class	Farmers and unskilled workers = 0	66.7	1	6	1.342
		Skilled workers and service = 1	18.6			
		Clerical staff = 2	3.7			
		Junior intellectuals = 3	3.1			
		Senior intellectuals = 4	3.0			
		Administrators and managers = 5	4.9			
	Social stratum	Lower class = 0	85.3	0	2	0.577
		Middle class = 1	6.8			
		Upper class = 2	7.9			
	Sports participation	Non-participant = 0	80.3	0	1	0.398
		Participant = 1	19.7			
Dependent variable	Self-rated Health^b^	1 = Poor; 2 = Fair; 3 = Good; 4 = Excellent	100.0 (3.0)	1	4	0.692

N_ID = 1,532, Total observations = 3,741.

a Indicates that the data in parentheses represent the mean age of 1,532 individuals, in years.

b Indicates that the data in parentheses represent the mean self-rated health score of 1,532 individuals, in points.

The lower social class comprises farmers and unskilled workers and skilled workers and service industry workers, the middle social class comprises clerical staff and junior intellectuals, and the upper social class comprises senior intellectuals and administrators and managers. Percentages are based on the analytic sample across person-wave observations unless otherwise indicated. For continuous variables (Age and SRH), values are presented as 100.0 (mean) in the Percentage/% column to indicate complete non-missingness (100.0%) and the mean shown in parentheses; SD is reported in the last column. SRH ranges from 1 (Poor) to 4 (Excellent).

### 2.3 Model construction

Based on the research questions, the model construction not only considers the two-dimensional effects of age and social class, as well as age and sports participation on health, but also focuses on analyzing the multidimensional and complex effects of age, social class, and sports participation on health. Furthermore, given the use of the CHNS longitudinal survey database, the data collected are longitudinal in nature. This means that participants’ information is repeatedly observed and recorded across different years, embedding each individual’s survey status at specific time points within their personal trajectory. Consequently, the data exhibit a hierarchical structure. Therefore, this study employs a hierarchical growth curve model to address the temporal variation of individual data within the longitudinal dataset. This model not only enables the analysis of health changes within and between individuals, but also accommodates multiple observations per subject. This maximizes the utilization of longitudinal data to validate previously proposed research hypotheses.

The model consists of one pair of submodels:

The first-level model captures the heterogeneous trajectories of self-rated health changes with age across individuals.

The second-level model reflects the variability in health change patterns between individuals.

The specific form of the first-level model is shown in Equation 1:

$$\begin{array}{l} \text{Healt}h_{\mathrm{ii}}=\pi_{0i}+\pi_{1i}\mathrm{Ag}e_{\mathrm{ti}}+\pi_{2i}\text{Spor}t_{\mathrm{ti}}+\pi_{3i}\text{Sport}\times \\ \mathrm{Ag}e_{\mathrm{ti}}+\sum_{j=1}^{M}\pi_{\mathrm{ji}}{\left(X_{j}\right)}_{\mathrm{ti}}+e_{\mathrm{ti}} \end{array}$$ (1)

The second-level model includes an intercept parameter model and a slope parameter model. The specific form of the intercept parameter model is shown in Equation 2:

$$\pi_{0i}=\beta_{00}+\beta_{01}\text{Stratu}m_{i}+\sum_{j=1}^{M}\beta_{0j}{\left(Z_{j}\right)}_{i}+\Gamma_{0i}$$ (2)

The specific forms of the slope parameter models are shown in Equations 3–5:

$$\pi_{1i}=\beta_{10}+\beta_{11}\text{Stratum}+\Gamma_{1}$$ (3)

$$\pi_{2i}=\beta_{20}+\beta_{21}\text{Stratu}m_{i}$$ (4)

$$\pi_{3i}=\beta_{30}+\beta_{31}\text{Stratu}m_{i}$$ (5)

The two-layer model is merged to form a stratified model for healthy growth curves, with its specific form shown in Equation 6:

$$\begin{array}{l} \text{Healt}h_{\mathrm{ti}}=\beta_{00}+\beta_{01}\text{Stratu}m_{i}+\beta_{10}\mathrm{Ag}e_{\mathrm{ti}}+\beta_{11}\text{Stratu}m_{i}\times \mathrm{Ag}e_{\mathrm{ti}}+\beta_{20}\text{Spor}t_{\mathrm{ti}}+\\ \beta_{21}\text{Stratu}m_{i}\times \text{Spor}t_{\mathrm{ti}}+\beta_{30}\mathrm{Ag}e_{\mathrm{ti}}\times \text{Spor}t_{\mathrm{ti}}+\beta_{31}\text{Stratu}m_{i}\times \\ \mathrm{Ag}e_{\mathrm{ti}}\times \text{Spor}t_{\mathrm{ti}}+\sum_{j=1}^{M}\beta_{0j}{\left(Z_{j}\right)}_{i}+(\Gamma_{0i}+\Gamma_{1i}\mathrm{Ag}e_{\mathrm{ti}}+e_{\mathrm{ti}}) \end{array}$$ (6)

In the model: Health represents an individual’s self-rated health status; *i* denotes the individual in the sample survey; *t* indicates the survey time; Age represents the individual’s age (centered by subtracting the mean of 18.60274, without rounding). After centering age, the model’s intercept term gains clearer practical significance, representing the health level of individuals at the average age, facilitating interpretation; Sport denotes the individual’s level of sports participation; Stratum indicates the individual’s social stratum; *M* represents the number of control variables; *Zj* denotes control variables that do not vary over time, such as household registration, region, gender, etc.; βpq (β00, β01, β10, β11, β20, β21, β30, β31, etc.) are fixed-effects model parameters; Γ0i and Γ1i represent the random effects for intercept and linear slope, respectively, following a mean-zero normal distribution. Together with the residuals (individuals over time), they form the random effect variance.

Additionally, to examine the stability of social class during adolescence (ages 12–18), this study constructed two multilevel linear models. First, a random intercept model (Equations 7, 8) was established to examine whether social class exhibits a significant overall trend of change over time. Building upon this, a random intercept and random slope model (Equations 9–11) was further developed. This model serves two critical functions: it tests whether significant heterogeneity exists in the rate of social class change among individuals, and it verifies whether the time-point effect remains insignificant even when accounting for differences in inter-individual change rates.

The first model is the random intercept model.

The specific form of the Level 1 model is shown in Equation 7:

$$\text{Stratu}m_{\mathrm{ti}}=\eta_{0i}+\eta_{1i}\mathrm{Wav}e_{\mathrm{ti}}+\varepsilon_{\mathrm{ti}}$$ (7)

The second-level model comprises an intercept parameter model and a slope parameter model. The specific form of the intercept parameter model is shown in Equation 2:

$$\eta_{0i}=\gamma_{00}+\zeta_{0i}$$ (8)

Stratum *ti* denotes the social stratum of individual *i* at time *t*, Wave *ti* represents the time-specific variable at the survey point, *η*_0*i*_ is the random intercept for individual *i*, *η*_1_ is the fixed effect for the time point, *ε_ti_* is the residual term for the first-level model, and *ζ*_0*i*_ is the random intercept for the second-level model.

The second model is a random intercept and random slope model, with its specific form shown in Equations 9–11:

First-level model:

$$\text{Stratu}m_{\mathrm{ti}}=\eta_{0i}+\eta_{1i}\mathrm{Wav}e_{\mathrm{ti}}+\varepsilon_{\mathrm{ti}}$$ (9)

Second-level model:

$$\eta_{0i}=\gamma_{00}+\zeta_{0i}$$ (10)

$$\eta_{1i}=\gamma_{10}+\zeta_{1i}$$ (11)

Among these, *η*_1*i*_ represents the random slope for individual *i*, *γ*10 denotes the fixed effect for the time point, and *ζ*_0*i*_ and ζ_1*i*_ are the random effects in the second-level model.

## 3 Results

### 3.1 Reality of health inequality across social classes

Univariate analysis of variance results based on five survey waves revealed statistically significant differences in self-rated health across social-class groups, with a pronounced trend toward widening health inequality over time (Table 2). In 1997, differences in self-rated health between the other social groups and Farmers and Unskilled Workers (2.974 ± 0.572) were not statistically significant, with scores ranging from 2.984 to 3.143 across all groups. By 2000, significant differences (*p* < 0.05) emerged for Junior Intellectuals (3.222 ± 0.592) and Senior Intellectuals (3.216 ± 0.780) relative to Farmers and Unskilled Workers (2.998 ± 0.683), marking the initial emergence of health inequality. By 2004, the health gap between Administrators and Managers (3.185 ± 0.689) and Farmers and Unskilled Workers (2.940 ± 0.724) widened further (*p* < 0.01). By 2006, health inequality continued to widen, with more occupational groups showing significant advantages over Farmers and Unskilled Workers (2.935 ± 0.718), including Clerical Staff (3.224 ± 0.647, *p* < 0.05), Junior Intellectuals (3.183 ± 0.661, *p* < 0.05), and Administrators and Managers (3.175 ± 0.633, *p* < 0.05).

Notably, the 2015 data revealed a further widening of health inequality. Except for Farmers and Unskilled Workers (2.769 ± 0.735), all other social-class groups showed significant differences: Skilled Workers and Service Workers (2.908 ± 0.706, *p* < 0.001), Clerical Staff (3.138 ± 0.708, *p* < 0.001), Junior Intellectuals (3.094 ± 0.669, *p* < 0.001), Senior Intellectuals (3.116 ± 0.686, *p* < 0.001), and Administrators and Managers (3.112 ± 0.714, *p* < 0.001). This pattern indicates a persistent widening of health inequality over the survey period.

**Figure 2:**
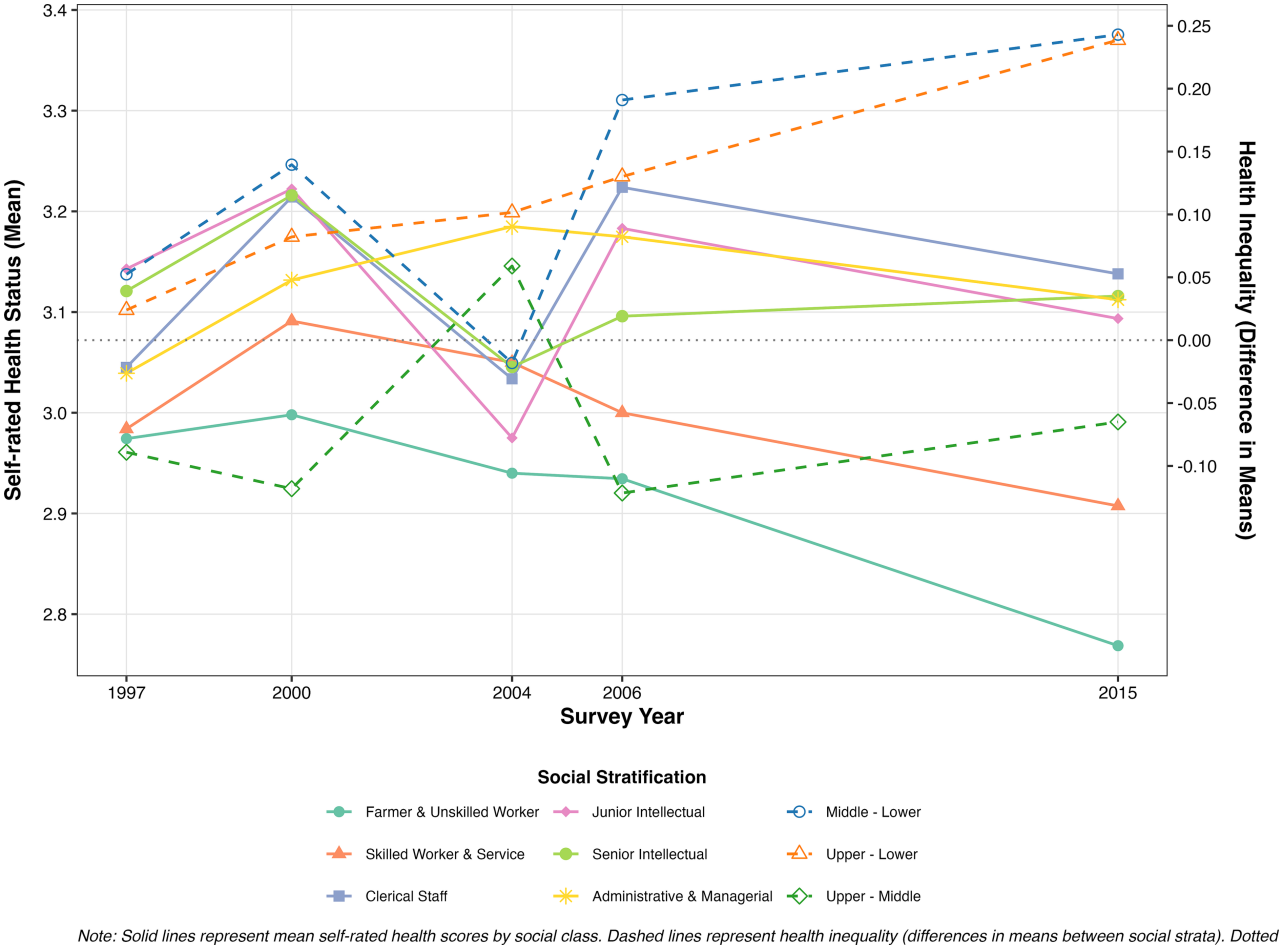
Trends in health status and health inequality by social stratification.

As shown in Figure 2, health levels across social strata exhibited a typical gradient structure: higher social status was associated with better health, and this gradient strengthened over time. High-tier groups (e.g., Administrators and Managers and Senior Intellectuals) maintained relatively stable and high health levels across all survey waves, whereas low-tier groups (Farmers and Unskilled Workers) exhibited pronounced health disadvantages with limited improvement from 1997 to 2015. Notably, the health trajectories of middle-tier groups (e.g., Clerical Staff and Junior Intellectuals) fluctuated between 2000 and 2015, indicating that the health advantage of the middle class was not stable and may have been shaped by more complex economic and social structural changes. This pattern suggests that health benefits are unevenly distributed across social strata and that the impact of socioeconomic status on health accumulates and intensifies over time.

These findings suggest that over time, health inequalities across social strata not only persist but are markedly widening. However, validating these hypotheses requires incorporating a life course perspective to explore the independent impact of social stratum on health while controlling for additional variables.

**Table 2:** Comparison of self-rated health scores across social classes.

Year	Social class	Score	95%CI
1997	Farmer and unskilled worker class	2.9743 ± 0.5722	2.9509, 2.9977
1997	Skilled worker and service class	2.9840 ± 0.6076	2.9305, 3.0374
1997	Clerical staff class	3.0450 ± 0.5115	2.9488, 3.1413
1997	Junior intellectual class	3.1429 ± 0.5690	3.0243, 3.2614
1997	Senior intellectual class	3.1209 ± 0.6966	2.9758, 3.2659
1997	Administrative and managerial class	3.0392 ± 0.6325	2.9612, 3.1172
2000	Farmer and unskilled worker class	2.9980 ± 0.6832	2.9633, 3.0327
2000	Skilled worker and service class	3.0911 ± 0.7125	3.0216, 3.1607
2000	Clerical staff class	3.2143 ± 0.6026	3.0835, 3.3451
2000	Junior intellectual class	3.2222 ± 0.5916*	3.0914, 3.3530
2000	Senior intellectual class	3.2159 ± 0.7798*	3.0507, 3.3811
2000	Administrative and managerial class	3.1317 ± 0.6636	3.0304, 3.2331
2004	Farmer and unskilled worker class	2.9400 ± 0.7244	2.9031, 2.9769
2004	Skilled worker and service class	3.0500 ± 0.6919	2.9820, 3.1180
2004	Clerical staff class	3.0337 ± 0.7300	2.8799, 3.1875
2004	Junior intellectual class	2.9750 ± 0.7111	2.8167, 3.1333
2004	Senior intellectual class	3.0455 ± 0.7414	2.8884, 3.2025
2004	Administrative and managerial class	3.1849 ± 0.6885**	3.0599, 3.3099
2006	Farmer and unskilled worker class	2.9345 ± 0.7184	2.8933, 2.9756
2006	Skilled worker and service class	3.0000 ± 0.7045	2.9311, 3.0689
2006	Clerical staff class	3.2239 ± 0.6472*	3.0660, 3.3817
2006	Junior intellectual class	3.1831 ± 0.6614*	3.0266, 3.3396
2006	Senior intellectual class	3.0959 ± 0.6701	2.9395, 3.2522
2006	Administrative and managerial class	3.1748 ± 0.6329*	3.0511, 3.2984
2015	Farmer and unskilled worker class	2.7687 ± 0.7350	2.7340, 2.8033
2015	Skilled worker and service class	2.9075 ± 0.7064***	2.8600, 2.9549
2015	Clerical staff class	3.1379 ± 0.7079***	3.0320, 3.2439
2015	Junior intellectual class	3.0935 ± 0.6691***	2.9813, 3.2057
2015	Senior intellectual class	3.1159 ± 0.6864***	3.0100, 3.2217
2015	Administrative and managerial class	3.1122 ± 0.7144***	3.0116, 3.2129

N_ID = 8,301, Total observations = 13,067; Data are presented as mean ± standard deviation. * indicates *P* < 0.05, ** indicates *P* < 0.01, *** indicates *P* < 0.001, compared with Farmer and unskilled worker class.

### 3.2 Health disparities between non-physical activity and physical activity groups

**Figure 3:**
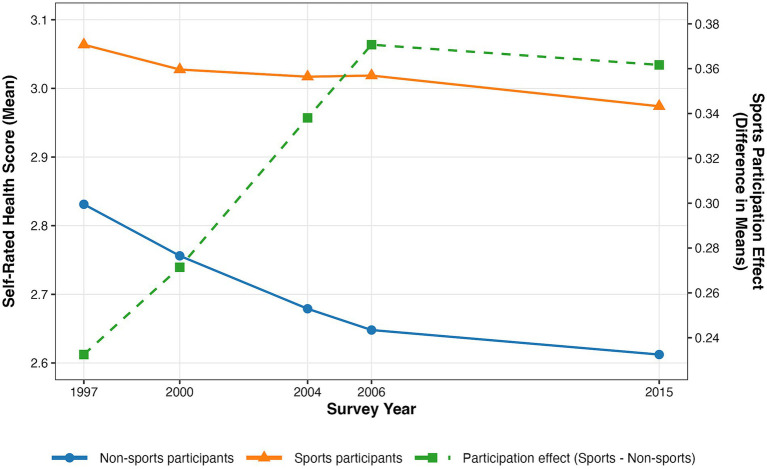
Trends in health status and health inequality by social participation stratification.

Comparisons of self-rated health scores between non-physical activity and physical activity groups (Table 3) reveal that, across all survey years, the physical activity group consistently reported better health status than the non-physical activity group, with statistically significant differences (*p* < 0.001). Simultaneously, as depicted in Figure 3, health levels exhibited a gradual decline over time regardless of physical activity participation. However, since the decline was smaller among the physically active group than among the inactive group, the health gap between them showed an increasing trend year by year. This finding provides an opportunity to incorporate physical activity behavior into the life course perspective to explore its impact on health, suggesting that physical activity participation likely plays a crucial moderating role in the gradual decline of health levels with age. Validating this hypothesis requires further investigation while controlling for additional variables.

**Table 3:** Comparison of self-rated health scores between participants with or without sports.

Year	Survey group	Score	95%CI
1997	Non-sports participants	2.8312 ± 0.7019	2.8181, 2.8442
1997	Sports participants	3.0637 ± 0.6368***	2.9992, 3.1281
2000	Non-sports participants	2.7561 ± 0.7765	2.7409, 2.7713
2000	Sports participants	3.0275 ± 0.6949***	2.9592, 3.0958
2004	Non-sports participants	2.6790 ± 0.8043	2.6635, 2.6944
2004	Sports participants	3.0170 ± 0.6642***	2.9526, 3.0814
2006	Non-sports participants	2.6480 ± 0.7958	2.6325, 2.6635
2006	Sports participants	3.0187 ± 0.6659***	2.9456, 3.0918
2015	Non-sports participants	2.6123 ± 0.7915	2.5994, 2.6252
2015	Sports participants	2.9740 ± 0.7083***	2.9261, 3.0218

N_ID: 26907, Total observations: 58,276; Data are presented as mean ± standard deviation; *** indicates *P* < 0.001, compared with non-sports participants.

### 3.3 Analysis results of the healthy growth curve model

The above findings provide two fundamental premises for examining whether childhood sports participation can overcome class-based health inequalities from a life course perspective.

Additionally, to examine the stability of social class during adolescence (ages 12–18), this study constructed two multilevel linear models. Results indicate that in the random intercept model, the survey time point did not significantly predict social class (η1 = 0.0001, *p* > 0.05); In the model incorporating random slope, the time-point effect remained insignificant (γ10 = 0.0001, *p* > 0.05). This indicates that during the critical developmental stage of 12–18 years, individuals’ relative socioeconomic status remains relatively stable and does not undergo systematic changes with age. This supports the theoretical rationale for treating socioeconomic status as a relatively stable variable in subsequent analyses.

Based on this, this study contextualized the above phenomena within individuals’ life courses (as they age). By constructing a healthy growth curve model and progressively incorporating interaction pathways—such as socioeconomic status × age → health, socioeconomic status × sports participation → health, sports participation × age → health, and socioeconomic status × sports participation × age → health—the research questions were thoroughly explored and addressed. Results are presented in Table 4.

**Table 4:** Analysis results of healthy growth curve model.

Variable	Model 1	Model 2	Model 3	Model 4	Model 5
Fixed effects					
Intercept	2.8258 ± 0.0271***	2.8256 ± 0.0271***	2.8256 ± 0.0272***	2.8259 ± 0.0272***	2.8275 ± 0.0272***
Area	0.1240 ± 0.0114***	0.1240 ± 0.0114***	0.1240 ± 0.0114***	0.1240 ± 0.0114***	0.1236 ± 0.0114***
Residency status	−0.0898 ± 0.0254***	−0.0897 ± 0.0254***	−0.0897 ± 0.0254***	−0.0901 ± 0.0255***	−0.0905 ± 0.0254***
Gender	0.0567 ± 0.0244*	0.0567 ± 0.0244*	0.0567 ± 0.0244*	0.0567 ± 0.0244*	0.0558 ± 0.0244*
Age (centered)	−0.0118 ± 0.0024***	−0.0119 ± 0.0025***	−0.0119 ± 0.0025***	−0.0121 ± 0.0025***	−0.0124 ± 0.0025***
Social stratum	0.0828 ± 0.0210***	0.0835 ± 0.0212***	0.0831 ± 0.0247***	0.0833 ± 0.0247***	0.0812 ± 0.0247**
Sports participation	−0.0312 ± 0.0298	−0.0305 ± 0.0299	−0.0309 ± 0.0326	−0.0088 ± 0.0690	0.0623 ± 0.0751
Social stratum × age		0.0012 ± 0.0042	0.0012 ± 0.0047	0.0011 ± 0.0047	0.0036 ± 0.0048
Social stratum × sports participation			0.0012 ± 0.0475	0.0002 ± 0.0476	−0.1900 ± 0.0932*
Sports participation × age				0.0056 ± 0.0155	0.0240 ± 0.0173
Social stratum × sports participation × age					−0.0526 ± 0.0222*
Random effects—variance components					
Level 1: Within-individual	0.6412 ± 0.0641***	0.6411 ± 0.0641***	0.6412 ± 0.0641***	0.6413 ± 0.0641***	0.6408 ± 0.0641***
Level 2: Intercept	0.2046 ± 0.0205***	0.2047 ± 0.0205***	0.2048 ± 0.0205***	0.2045 ± 0.0204***	0.2035 ± 0.0204***
Linear growth rate	0.0058 ± 0.0006***	0.0064 ± 0.0006***	0.0064 ± 0.0006***	0.0065 ± 0.0007***	0.0077 ± 0.0008***
Model fit					
AIC	7648.042	7649.969	7651.968	7653.835	7650.192
BIC	7716.540	7724.694	7732.920	7741.014	7743.599

N_ID = 1,532, Total observations = 3,741. Data are presented as mean ± SE. * indicates *P* < 0.05, *** indicates *P* < 0.001. AIC, Akaike information criterion; BIC, Bayesian information criterion.

After controlling for demographic characteristics, the impact of social class on health status was examined. Model 1 served as the baseline model, incorporating only core independent variables such as age and social class. Results indicated that age (centered) exerted a significant negative effect on health status (β₁₀ = −0.0118, *p* < 0.001), suggesting a marked decline in health levels with increasing age. Social class, however, exhibited a significant positive predictive effect (β₀₁ = 0.0828, *p* < 0.001), meaning higher social class groups generally reported better health status than lower social class groups. This further confirms the existence of class inequality in health (partially supporting H1, while H2 is not supported).

Models 2 through 4 sequentially introduced second-order interaction terms. Results showed that the interaction effects of social class × age and social class × sports participation were not significant. The interaction term for sports participation × age also failed to reach significance, suggesting that simple binary interactions may be insufficient to explain the complex mechanisms among variables. Ultimately, a statistically significant moderation effect was identified in Model 5, which included the third-order interaction term of social class × sports participation × age. Regarding model fit, Model 5’s AIC value was 7650.192, slightly higher than the baseline Model 1 (7648.042). However, the difference was close to 2, indicating no significant distinction between the two models. More importantly, Model 5 demonstrated significant second- and third-order interaction effects (*p* < 0.05), a finding of substantial theoretical value. Given this study’s focus on exploring complex relationships rather than pursuing model parsimony, Model 5 was selected as the final model.

**Figure 4:**
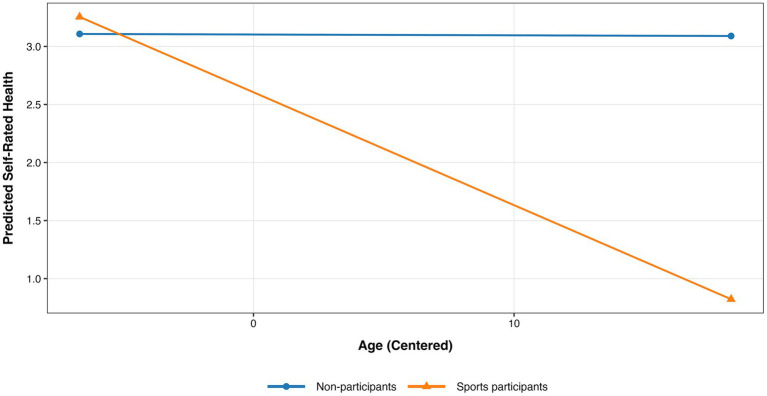
Interaction between sports participation and age on self-rated health in middle social stratum.

The third-order interaction term coefficient in this model is significant (β31 = −0.0409, *p* < 0.05). This finding indicates that for groups from different socioeconomic strata, if their levels of sports participation differ, their health status will exhibit complex, differential changes with age—that is, heterogeneity exists (validating H3). As such complex dynamics are difficult to discern directly from Table 4, this study categorized occupational strata into three groups (lower social class, middle social class, and upper social class) and constructed stratified health growth models for each group. Results are presented in Table 5 (control variables were accounted for but omitted for brevity and clarity). For the lower socioeconomic group, age exhibits a significant negative effect on self-rated health (β10 = −0.0127 ± 0.0025, *p* < 0.001), indicating a pronounced decline in health status with age among individuals in lower socioeconomic strata. The main effect of sports participation was not significant, and the interaction term between sports participation and age also failed to reach statistical significance (β30 = 0.0275 ± 0.0176, *p* > 0.05). This pattern indicates that among lower socioeconomic groups, although sports participation shows a weak protective trend (partially supporting H4), it fails to significantly buffer the negative impact of age on health, with health inequalities persisting throughout the life course. For middle socioeconomic groups, the main effect of age was insignificant, suggesting relatively stable health levels with age across this stratum. However, sports participation exhibited a significant negative main effect (β20 = −0.4974 ± 0.2298, *p* < 0.05), while the interaction term between sports participation and age showed a marginally significant negative trend (β30 = −0.0965 ± 0.0517, *p* ≈ 0.06). Further analysis of this interaction revealed a marginally significant decline in health among sports participants with increasing age, while no significant change was observed among non-participants (see Figure 4). This finding may reflect the health selection effect—where individuals with relatively poorer health in the middle class are more inclined to engage in sports activities to improve their health, resulting in a lower baseline health level among sports participants. For the upper socioeconomic group, neither the age effect, the main effect of sports participation, nor their interaction reached statistical significance. This indicates that health levels among this group remain relatively stable, with sports participation showing no significant moderating effect. This may stem from the group’s already abundant access to health resources and protective factors (H5 holds; H6 and H7 not supported).

**Table 5:** Effect of sports participation on health in different social-class participants.

Variables	Lower stratum	Middle stratum	Upper stratum
Control_Variables	Controlled	Controlled	Controlled
Age (centered)	−0.0127 ± 0.0025***	−0.0007 ± 0.0105	−0.0022 ± 0.0109
Sports participation	0.0798 ± 0.0765	−0.4974 ± 0.2298*	−0.1860 ± 0.1653
Sports participation × age	0.0275 ± 0.0176	−0.0965 ± 0.0517	−0.0603 ± 0.0408
Model fit			
AIC	6586.663	556.658	625.125
BIC	6653.412	595.612	665.682

Lower stratum *n* = 1,306 (observations = 3,191), Middle stratum *n* = 109 (observations = 255), Upper stratum *n* = 133 (observations = 295); Data are presented as mean ± standard error; * indicates *P* < 0.05, *** indicates *P* < 0.001; Lower stratum includes farmers and non-skilled workers, technical workers and service industry workers; Middle stratum includes clerical staff, junior and intermediate intellectuals; Upper stratum includes senior intellectuals, administrative officials and enterprise managers; AIC, Akaike Information Criterion; BIC, Bayesian Information Criterion.

## 4 Discussion

This study systematically examined the complex relationship between social class, sports participation, and healthy aging from a life course perspective by constructing a multilevel growth curve model based on five waves of longitudinal survey data. Key findings can be summarized in three aspects: First, social class inequality in health exhibits significant structural rigidity, manifested as persistence across time and widening gradients; Second, the health benefits of physical activity participation exhibit pronounced class heterogeneity. Third, a significant three-way interaction exists among social class, physical activity participation, and age, indicating that the impact of physical activity participation on healthy aging is strongly moderated by an individual’s social class background. These findings will be elaborated upon below, drawing on relevant theories and existing research.

### 4.1 Structural solidification of health inequality and life course differentiation mechanisms

This study reveals that from 1997 to 2015, health disparities across China’s social strata significantly widened, particularly with the health gradient between the middle and lower classes rising from 0.11 to 0.26. This finding indicates that health inequality is not a transient social phenomenon but exhibits profound structural solidification. This trend is closely linked to the macroeconomic context of China’s socioeconomic transformation. While deepening market reforms have driven overall economic growth, they may have exacerbated inequalities in resource allocation, including access to health resources (36). Higher-status occupational groups (e.g., Administrators and Managers and Senior Intellectuals) leverage their socioeconomic advantages to access superior healthcare services, health information, and healthier living environments, thereby better maintaining their health status. Conversely, lower-tier groups like Farmers and Unskilled Workers face multiple pressures—resource constraints, occupational exposure, and accumulated health risks—trapping them at the bottom of the health hierarchy.

After controlling for demographic variables, the growth curve model further revealed that social class exerts a stable positive predictive effect on health (β01 = 0.0828, *p* < 0.001). This finding aligns with the Fundamental Cause Theory of health stratification (37), which posits that socioeconomic status acts as a foundational determinant of health by influencing multiple risk exposures and access to adaptive resources. Furthermore, age exhibits a significant negative predictive effect on health (β10 = −0.0118, *p* < 0.001). However, the interaction term between age and socioeconomic status was not significant. This indicates that the structural entrenchment of health inequalities occurs through a life course differentiation mechanism: individuals across socioeconomic strata age synchronously along two or more distinct health trajectories that start at different points but converge at similar slopes. The health gap between strata forms early in the life course and is fully maintained throughout the subsequent aging process. This implies that health risks and resource constraints faced by lower-class individuals in early life lead to the long-term entrenchment of their health disadvantage through this life course differentiation mechanism. This pattern strongly suggests that the key mechanisms driving health disparities likely occur primarily in the early stages of the life course. Once this health stratification pattern, determined by social class, is established in early life, it persists as a structural imprint, remaining relatively stable throughout the subsequent life course. This finding provides crucial empirical evidence for understanding the production and reproduction mechanisms of health inequalities in contemporary Chinese society.

### 4.2 Class differentiation in the health effects of physical activity participation: a life course

The most significant theoretical contribution of this study lies in revealing the pronounced class heterogeneity in the health effects of sports participation. By introducing a three-way interaction term of social class × sports participation × age, which proved statistically significant (β31 = −0.0526, *p* < 0.05), we confirmed that the health returns from sports participation are not homogeneous but are heavily constrained by an individual’s socioeconomic background.

Among lower socioeconomic groups, neither the main effect of physical activity participation nor its interaction with age reached statistical significance. This finding supports the “structural constraints” theory (38). For individuals in resource-constrained lower socioeconomic strata, health is primarily dominated by structural factors such as poverty, harsh working conditions, and inadequate healthcare resources (39). Notably, this group exhibited a significant age-related decline in health (β10 = −0.0127 ± 0.0025, *p* < 0.001), further illustrating that their health trajectory is constrained by powerful structural pressures. This implies that physical activity participation alone is insufficient to effectively counteract formidable structural health risks. This finding suggests that health promotion policies targeting vulnerable groups will yield extremely limited results if they focus solely on advocating physical exercise while neglecting the underlying socioeconomic hardships.

Among the middle-stratum population, the observed “health-behavior paradox” should not be interpreted as evidence that adolescent sports participation is inherently detrimental. Rather, it more likely reflects a class-conditioned erosion of the long-term health returns to early sports participation. Existing research suggests that physical activity in adolescence can promote continued activity in adulthood, but this tracking is far from deterministic, and overall activity levels tend to decline from adolescence to adulthood (40, 41). In this sense, adolescent sports participation constitutes an early health asset, but whether this asset is converted into long-term health advantage depends heavily on later-life social conditions. Middle-stratum individuals may be especially vulnerable to such conversion failure. Compared with lower-stratum groups, they are more likely to possess health awareness and early participation opportunities; yet compared with upper-stratum groups, they often face stronger job strain, persistent rushing, greater work–family spillover, fragmented leisure time, and insufficient recovery resources. Prior studies show that job strain predicts later leisure-time physical inactivity (42), that rushing is associated with poorer self-rated and mental health (43), and that leisure-time physical activity can buffer work–family spillover only when adequate recovery is available (44). Moreover, the literature on the physical activity paradox suggests that the health implications of physical activity are domain-specific rather than uniform (45). Accordingly, the steeper health decline observed here is better understood not as a harmful effect of adolescent sports participation itself, but as evidence that the long-term health returns to early sports participation are more likely to be weakened or eroded among middle-stratum individuals under later-life pressure and recovery constraints.

Among the upper socioeconomic groups, physical activity participation showed no significant moderating effect. This finding may relate to the “Resource Substitution Theory.” Privileged groups typically possess diverse health-protective resources (e.g., high-quality healthcare, strong health literacy, low-stress work environments), which effectively buffer age-related health decline. Consequently, the marginal health benefits of physical activity participation become relatively insignificant (46).

In summary, the findings of this study reveal how health inequalities become further complicated through the behavioral mechanism of physical activity participation across the life course. Different socioeconomic groups not only start from different health baselines but also exhibit systemic differences in their capacity to translate health behaviors into health outcomes. These differences ultimately manifest as a dynamic evolution of health inequalities through a three-level interaction.

### 4.3 Theoretical integration and policy implications

The findings of this study offer significant insights for understanding the mechanisms that produce health inequalities and for formulating relevant public health policies.

At the theoretical level, our research advances the interdisciplinary integration of health sociology and sports science. Results indicate that sports participation cannot be simplistically viewed as an independent form of “health capital”; its benefits are highly contingent upon an individual’s position within the social structure. This challenges the prevailing “individualistic” paradigm in health promotion, emphasizing the necessity of understanding health behaviors within broader socioeconomic contexts. Our findings align with theoretical dialogs on “Resource Reinforcement” and “Resource Substitution,” indicating differing patterns of relationship between physical activity participation and existing health resources across socioeconomic strata (47).

At the policy level, health promotion should move beyond universal exhortations to exercise and adopt a stratified intervention framework that combines structural support with behavior-specific guidance. For lower-class groups, the priority is not merely to increase participation, but to reduce the structural barriers that prevent sports participation from being converted into meaningful health gains. This requires not only expanding low-cost or free access to community- and school-based exercise programs, but also improving facility accessibility, schedule flexibility, occupational health protection, and the integration of basic health screening with exercise services. For middle-class groups, the policy focus should shift from simple participation promotion to the maintenance of long-term health returns. Given their vulnerability to job strain, work-family pressure, fragmented leisure time, and insufficient recovery, interventions should emphasize time-compatible exercise arrangements, workplace health promotion, recovery management, injury prevention, and professionally guided exercise prescriptions. For upper-class groups, by contrast, since the marginal health return of additional sports participation appears to be relatively limited, policy should place less emphasis on expanding participation volume and more on optimizing exercise quality, long-term adherence, and balanced health management. In this sense, class-sensitive interventions should not be understood merely as different levels of encouragement across social groups, but as targeted policy arrangements that reduce class-based barriers and strengthen each group’s capacity to translate sports participation into lasting health benefits. To facilitate policy uptake, these findings should also be translated into policy briefs and practice-oriented evidence summaries for education, health, and sports authorities, and communicated through academic–policy dialog and advisory channels.

### 4.4 Research limitations and future directions

This study has several limitations. First, although self-rated health is widely used in population-health research, it remains a subjective measure and may be influenced by reporting heterogeneity across social groups. Future studies should complement it with objective indicators, such as diagnosed conditions, biomarkers, or physical fitness measures. Second, because the study spans an 18-year follow-up period, attrition and selective survival may have introduced selection bias. Participants with poorer health or greater socioeconomic disadvantage may have been less likely to remain under observation, which means that later-life health inequalities may be conservatively estimated. Third, sports participation was measured in a relatively broad way and did not distinguish among activity type, intensity, frequency, or context. As a result, the present analysis can identify broad class-differentiated associations in sports participation overall, but cannot determine which specific forms of early sports participation are most strongly linked to long-term health trajectories.

In addition, the analysis is based on data up to 2015, which may limit the generalizability of the findings to more recent social and policy contexts.

Future research should address these limitations by using more refined measures of physical activity, attrition-adjusted longitudinal designs, and mechanism-oriented analyses capable of explaining how sports participation is translated into unequal health returns across social classes.

## 5 Conclusion

Drawing on growth-curve models and a life-course perspective, this study examined the interactive effects of social class, sports participation, and age on health. Three main conclusions emerge. First, health inequality displays clear structural consolidation in the context of social transformation. The health gradient between social classes widened markedly from 1997 to 2015 and followed an approximately parallel trajectory across the life course: disparities established early in life were largely preserved into later adulthood. Second, the health returns to physical activity are strongly class-differentiated and dynamically evolving. The significant three-way interaction among social class, physical activity, and age shows that the age profile of physical activity’s effect on healthy aging is fundamentally conditioned by social class position. For lower-class groups, structural constraints limit the ability to convert physical activity into health gains. Among middle-class groups, a “health behavior paradox” emerges, whereby higher levels of physical activity are associated with a steeper decline in health. For the upper class, by contrast, resource saturation attenuates additional benefits from physical activity. Third, these findings call for a re-examination of current health promotion policies. Strategies that rely solely on universal exhortations to exercise are insufficient to reduce health inequality and may even generate adverse effects in specific groups if class-specific resource endowments and structural constraints are ignored.

Future health promotion should undergo a dual transformation. Conceptually, policy must move beyond interventions that target individual lifestyles in isolation and acknowledge that the effectiveness of health behaviors is deeply embedded in social structural positions. Practically, it should integrate structural reforms with targeted behavioral interventions: alleviating structural barriers to health through improved working conditions and stronger social protection, while designing differentiated exercise and health-promotion programs aligned with the resources and needs of distinct socioeconomic groups. Only under such an integrated governance framework can the mechanisms reproducing health inequality be effectively mitigated in the pursuit of healthy aging.

During the preparation of this work the authors used ChatGPT in order to improve language. After using this tool, the authors reviewed and edited the content as needed and take full responsibility for the content of the publication.

## Data Availability

The raw data supporting the conclusions of this article will be made available by the authors, without undue reservation.
